# Comment on “48-Week Outcome after Cessation of Nucleos(t)ide Analogue Treatment in Chronic Hepatitis B Patient and the Associated Factors with Relapse”

**DOI:** 10.1155/2019/4927083

**Published:** 2019-01-01

**Authors:** Yafei Guo, Lingan Wang

**Affiliations:** ^1^Department of Hematology, The Second Affiliated Hospital of Fujian Medical University, Quanzhou 362000, China; ^2^Department of Pediatrics, Guangdong Women and Children's Hospital, Guangzhou 511442, China

We read with great interest the study conducted by Wen-xiong Xu et al. published in* Canadian Journal of Gastroenterology and Hepatology *[1]. The authors indicated that nucleos(t)ide analogues (NA) cessation is safe under supervision. Age and hepatitis B surface antigen (HBsAg) level can be predictive factors for virologic relapse. The data of the study demonstrated that, among 62 enrolled patients with chronic hepatitis B (CHB), a total of 23 patients (37.1%) experienced nonrelapse (Category A), while four patients (6.5%) remained with virologic relapse (Category B), 14 (22.6%) changed to nonrelapse before a transient virological relapse (Category C), and 21 (33.8%) were treated again with NAs due to clinical relapse (Category D). The study conducted by Xu et al. was elegantly designed and the results are very inspiring. However, the nonsustained response rate of up to 62.9% makes us a bit worried about long-term benefit for CHB patient after NAs cessation.

Whether and when CHB patients should be discontinued from NAs antiviral treatment after a sustained virological response is a dilemma. Although it is well established that the goal of treatment for chronic HBV infection is to improve quality of life and survival of the infected person by preventing progression of the disease to cirrhosis, decompensated cirrhosis, end-stage liver disease, hepatocellular carcinoma, and death [2], to achieve the therapeutic goals, HBV replication should be suppressed in a sustained manner, even if the patient's ALT level is within the upper limit of normal. Studies have revealed that although ALT levels are normal, liver fibrosis still exists in CHB patients and the disease is still in progress [3–5]. In the study of Xu et al. although category A was defined as no relapse, an increased HBV DNA viral load was still observed. The increasing trend of HBV DNA viral loads are more pronounced in Category B and Category C. These patients are at risk of sustained HBV replication even under long-term supervision, accompanied with re-ignition of hepatic inflammation, especially in certain specific situations such as the use of steroids. This re-ignition of liver inflammation will lead to further deterioration of liver fibrosis. Therefore, as recommended by the APASL guideline [2], the goal of CHB treatment can be achieved if HBV replication can be suppressed in a sustained manner. Then, the accompanying reduction in histological activity of CHB lessens the risk of cirrhosis and hepatocellular carcinoma, particularly in noncirrhotic patients. In CHB patients, as long as HBV DNA is positive, which means HBV continues to replicate, cessation of NAs may not be an option. Clinical guidelines have suggested that only HBsAg seroconversion is an ideal endpoint to NAs cessation [6, 7].

Another interesting result in the study is the relationship between age and HBsAg levels for sustained viral response. As confirmed by Xu et al.'s study and other similar studies [8–10], the younger the patient and the lower the serum HBsAg level, the higher the possibility of achieving a sustained virological response after NAs cessation. The reason may be the different immune status of patients. Chronic HBV is an infectious disease with a pathogenesis and course that depends on the virus-host interaction. A study has shown that the younger the patient, the higher the possibility to obtain virological response and HBsAg seroconversion after interferon treatment [11]. In particular, long-term HBV infection will significantly inhibit the immune system in CHB patients [12]. In addition, interferon as an add-on sequential regimen to tenofovir resulted in greater loss of HBsAg compared to tenofovir monotherapy [13]. Viral load reduction followed by immune modulation may be a potentially useful approach. Therefore, in Xu et al.'s study, for CHB patients with low-level HBsAg, it may be more effective to add interferon treatment as an immune modulation, rather than stop NAs, to further NAs cessation to further improve the possibility of HBsAg seroconversion and achieve a more suitable timing for NAs cessation. However, further prospective studies are required to confirm this.

Nevertheless, data from this study demonstrate that HBsAg and age are factors closely related to sustained virological response after NAs cessation. This is a valuable recommendation for real clinical practice. NAs cessation can be recommended in CHB patients with relatively younger age and relatively low serum HBsAg levels, to avoid adverse effects of long-term NAs, such as nephrotoxicity and Fanconi syndrome. CHB patients who are at a high risk of relapse after NAs cessation are not recommended to stop NAs antiviral treatment, thus avoiding patients self-discontinuing NAs treatment and the related unpredictable complications. This is especially so in the Asia-Pacific region, where NAs adherence is generally poor [14].
